# Effects of the number of neoadjuvant therapy cycles on clinical outcomes, safety, and survival in patients with metastatic colorectal cancer undergoing metastasectomy

**DOI:** 10.32604/or.2022.026659

**Published:** 2023-01-05

**Authors:** YUNG-SUNG YEH, HSIANG-LIN TSAI, YEN-CHENG CHEN, WEI-CHIH SU, PO-JUNG CHEN, TSUNG-KUN CHANG, CHING-CHUN LI, CHING-WEN HUANG, JAW-YUAN WANG

**Affiliations:** 1 Division of Trauma and Surgical Critical Care, Department of Surgery, Kaohsiung Medical University Hospital, Kaohsiung Medical University, Kaohsiung, Taiwan; 2 Department of Emergency Medicine, Faculty of Post-Baccalaureate Medicine, College of Medicine, Kaohsiung Medical University, Kaohsiung, Taiwan; 3 Graduate Institute of Injury Prevention and Control, College of Public Health, Taipei Medical University, Taipei, Taiwan; 4 Division of Colorectal Surgery, Department of Surgery, Kaohsiung Medical University Hospital, Kaohsiung Medical University, Kaohsiung, Taiwan; 5 Department of Surgery, Faculty of Medicine, College of Medicine, Kaohsiung Medical University, Kaohsiung, Taiwan; 6 Graduate Institute of Clinical Medicine, College of Medicine, Kaohsiung Medical University, Kaohsiung, Taiwan; 7 Department of Surgery, Faculty of Post-Baccalaureate Medicine, College of Medicine, Kaohsiung Medical University, Kaohsiung, Taiwan; 8 Graduate Institute of Medicine, College of Medicine, Kaohsiung Medical University, Kaohsiung, Taiwan; 9 Center for Cancer Research, Kaohsiung Medical University, Kaohsiung, Taiwan; 10 Pingtung Hospital, Ministry of Health and Welfare, Pingtung, Taiwan

**Keywords:** Metastatic colorectal cancer, Neoadjuvant chemotherapy/targeted therapy, Treatment cycles, Metastasectomy

## Abstract

The controversial outcomes in patients with metastatic colorectal cancer (mCRC) highlight the need for developing effective systemic neoadjuvant treatment strategies to improve clinical results. The optimal treatment cycles in patients with mCRC for metastasectomy remain undefined. This retrospective study compared the efficacy, safety, and survival of cycles of neoadjuvant chemotherapy/targeted therapy for such patients. Sixty-four patients with mCRC who received neoadjuvant chemotherapy/targeted therapy following metastasectomy were enrolled between January 2018 and April 2022. Twenty-eight patients received 6 cycles of chemotherapy/targeted therapy, whereas 36 patients received ≥7 cycles (median, 13; range, 7–20). Clinical outcomes, including response, progression-free survival (PFS), overall survival (OS), and adverse events, were compared between these two groups. Of the 64 patients, 47 (73.4%) were included in the response group, and 17 (26.6%) were included in the nonresponse group. The analysis revealed chemotherapy/targeted therapy cycle and pretreatment serum carcinoembryonic antigen (CEA) level as independent predictors of the response as well as overall survival and chemotherapy/targeted therapy cycle as an independent predictor of progression (all *p* < 0.05). Furthermore, our results revealed shorter operation time, lower estimated operative blood loss, higher response rate, lower progression rate, and higher survival rate in ≥7 cycles of chemotherapy/targeted therapy group (all *p* < 0.05), but no statistical differences in adverse events were observed between the two groups (all *p* > 0.05). The median OS and PFS were 48 months (95% CI, 40.855–55.145) and 28 months (95% CI, 18.952–37.48) in the ≥7-cycle group and 24 months (95% CI, 22.038–25.962) and 13 months (95% CI, 11.674–14.326) in the 6-cycle group, respectively (both *p* < 0.001). The oncological outcomes in the ≥7-cycle group were significantly better than those in the 6-cycle group, without significant increases in adverse events. However, prospective randomized trials are mandatory to confirm the potential advantages of cycle numbers of neoadjuvant chemotherapy/targeted therapy.

## Introduction

Colorectal cancer (CRC) is one of the leading cause of cancer-related death in Western populations and the third most common malignancy worldwide [1,2]. Despite early detection of CRC and considerable advancements in treatments that have improved survival, mortality and morbidity rates remain high, with distant metastases occurring in up to 50%–60% of patients [3,4]. Metastatic CRC (mCRC) typically develops metachronously after the treatment of locoregional CRC, with the liver being the most common site of involvement [3,5,6].

The median 5-year survival rate of patients with mCRC has increased from <10% to 35%–40%, whereas median overall survival (OS) has increased from <12 months to approximately 42 months [1,7]. The only potentially curative treatment is complete resection of metastatic lesions, but the outcomes remain poor. Accordingly, multimodality treatment approaches, including adjuvant or neoadjuvant chemotherapy, are implemented to improve outcomes and survival [8–11]. Some studies revealed the mCRC patients who received primary tumor resection and targeted therapy combination chemotherapy had better clinical outcomes than patients who did not receive primary tumor resection [12–14]. In particular, neoadjuvant chemotherapy/targeted therapy can improve patient survival after surgery for mCRC, including higher favorable progression-free survival (PFS) and OS without a significant toxicity increase [9,12], and therapeutic options for metastatic CRC (mCRC) have changed significantly in recent years [12].

Some studies have demonstrated no significant differences in morbidity or mortality when neoadjuvant chemotherapy/targeted therapy was used before metastectomy [15,16], whereas others have concluded that extended durations of neoadjuvant chemotherapy/targeted therapy are associated with worse perioperative outcomes after metastectomy [12,15–17]. This result could establish treatment protocols to allow appropriately selecting patients to develop precise treatment plans.

Patients with mCRC who undergo metastasectomy after different neoadjuvant treatment regimens exhibit outcomes that range between favorable and poor [6]. In this study, we compared patients with mCRC receiving 6 cycles of neoadjuvant chemotherapy/targeted therapy with those receiving ≥7 cycles of the neoadjuvant therapy. We hypothesized that ≥7 cycles might lead to improved efficacy and survival, with similar safety profiles. Our findings are expected to be useful in clinical decision-making in patients with mCRC scheduled for neoadjuvant therapy and major metastasectomy. The aim of our study is to determine the effect of cycles of neoadjuvant chemotherapy/targeted therapy on mCRC patients.

## Materials and Methods

### Patients and study design

This retrospective observational study included 64 patients with mCRC (including distant metastasis such as liver, lung, and ovary metastasis) who received neoadjuvant chemotherapy/targeted therapy and metastasectomy between January 01, 2018, and April 30, 2022, at Kaohsiung Medical University Hospital. This study was approved by the Institutional Review Board of Kaohsiung Medical University Hospital (KMUHIRB-E(I)-20200036). Baseline investigations consisted of a complete history review, physical examination, laboratory tests, pathological examination, and imaging (i.e., chest radiography, abdominal computed tomography [CT], and additional magnetic resonance imaging [MRI] if the CT scan could not clarify the cancer stage). TNM classification was determined according to the American Joint Committee on Cancer/Union for International Cancer Control criteria [3].

Other inclusion criteria were as follows: (1) having mCRC with pathologically confirmed adenocarcinoma, (2) being ≥20 years old, (3) having an Eastern Cooperative Oncology Group performance status of 0–2, and (4) having adequate hematological, renal, and liver function. We excluded patients with any of the following: central nervous system metastases, previous malignancy, infectious disease (for which neoadjuvant chemotherapy/targeted therapy is not acceptable), serious concurrent medical illness (i.e., clinically significant cardiac disease or liver disease), life expectancy <3 months, and inability to receive premetastasectomy neoadjuvant therapy. In patients with liver, lung, or ovary metastasis, metastasectomy was performed only if a good response to neoadjuvant therapy was obtained.

Clinicopathological characteristics, such as age, sex, chemotherapy/targeted therapy cycle numbers, targeted agents, pretreatment metastasis site, primary lesion location, *RAS* gene status, synchronous/metachronous metastasis, operation time, estimated operative blood loss, pretreatment serum carcinoembryonic antigen (CEA) level, posttreatment serum CEA level, type of targeted therapy, response status, progression status, survival status, and adverse events of grade III or more, were analyzed. In this study, we explored the efficacy, safety, and survival profile between preoperative cycle numbers (6 cycles *vs*. ≥7 cycles) of chemotherapy/targeted therapy in mCRC.

In this study, we compared the survival data of patients with mCRC receiving neoadjuvant chemotherapy/targeted therapy by using real-world data from one institution.

### Efficacy and safety outcome measures

The primary endpoints were the response rate, PFS, and OS. The secondary endpoints were adverse events during neoadjuvant chemotherapy/targeted therapy. Physical examination, liver and kidney function tests, complete blood count with differential count evaluation, and serum CEA level examinations were performed before treatment initiation and every 2 weeks thereafter. Abdominal or chest CT (depending on metastatic lesions) and additional MRI were performed every 2–3 months during chemotherapy/targeted therapy, and chest X-ray was performed annually. A bone scan or positron emission tomography scan was selectively performed to obtain images of suspicious findings at specific locations on CT or MRI images and suspicious metastases. All enrolled patients were followed up every 2–3 months until the last visit or death. The median follow-up time for all patients was 22 months (range: 8–48 months).

Responses were classified according to the Response Evaluation Criteria in Solid Tumors [16]. Complete remission (CR) was defined as the disappearance of all target cancer lesions. Partial response (PR) was defined as a ≥30% reduction of the sum of the longest diameters of metastatic lesions, with no signs of new lesions. Progressive disease (PD) was defined as a ≥20% cumulative increase in the longest diameter of the target lesion, and the smallest sum of the longest diameters recorded before the patient began treatment was used as a reference. Identification of one or more new lesions was also categorized as PD. The contraction rate of stable disease (SD) is was insufficient to meet the PR criteria, and the increase was not sufficient to meet the PD criteria [18]. PFS was defined as the interval between the start of neoadjuvant chemotherapy/targeted therapy and the first record of progression, regardless of the patient’s treatment status or final follow-up. OS was defined as the interval from the beginning of neoadjuvant chemotherapy/targeted therapy to the date of death or last visit [11,19]. Adverse events were monitored and graded in each cycle according to the National Cancer Institute–Common Terminology Criteria for Adverse Events (NCT-CTCAE) Version 4.3 (https://ctep.cancer.gov/protocoldevelopment/electronic_applications/ctc.htm).

### Statistical analysis

Continuous variables are presented as mean ± SD, and dichotomous variables are presented as numbers and percentage values. Categorical and continuous variables were analyzed using the Fisher exact test/chi-square test and the Mann–Whitney *U* test, respectively. Univariate and multivariate analyses were performed to evaluate independent predictors. Cox proportional hazard regression was performed to evaluate the independent predictors of progression and survival. PFS and OS were calculated and plotted according to the Kaplan–Meier method, and the log-rank test was used to compare time-to-event distribution. *p* < 0.05 was considered statistically significant for all tests. All statistical analyses were performed using the IBM Statistical Package for the Social Sciences, Version 21.0 (IBM Corp, Armonk, NY, USA).

## Results

### Patients’ population and disposition

**Figure 1:**
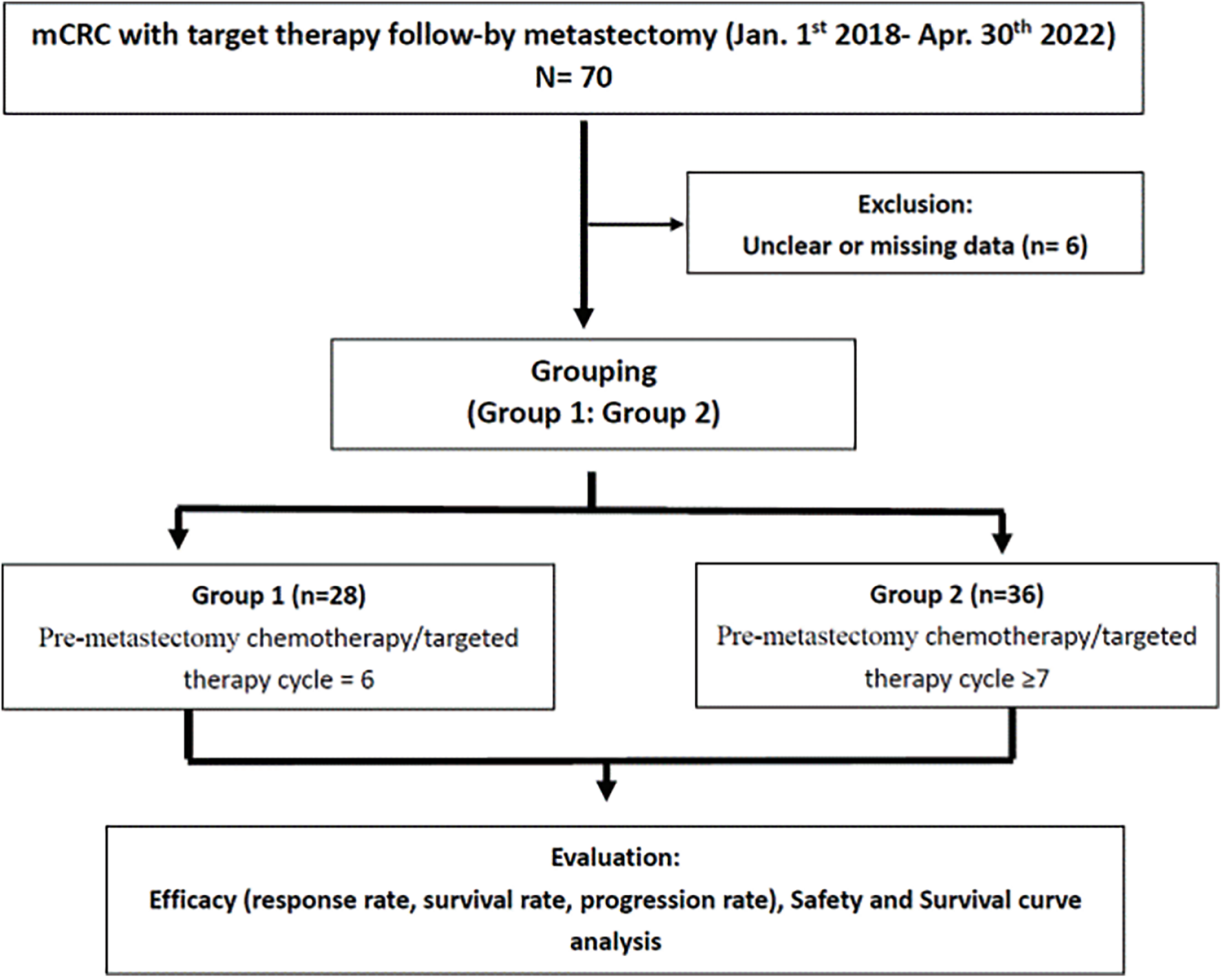
Consolidated Standards of Reporting Trials (CONSORT) flow diagram.

We enrolled 64 patients with mCRC (40 men and 24 women; mean age, 60 years; range 30–83 years) who received 6 or ≥7 cycles of neoadjuvant chemotherapy/targeted therapy followed by metastasectomy. Among them, 28 (43.8%) received 6 cycles (the 6-cycle group), and 36 (56.2%) received ≥7 cycles (the ≥7-cycle group; median: 13; range, 7–20) (Fig. 1).

The patients’ baseline characteristics, neoadjuvant therapy regimens, clinicopathological features, and tumor characteristics as well as efficacy and safety data are listed in Table 1. The neoadjuvant chemotherapy regimen is FOLFIRI (5-fluorouracil + leucovorin + irinotecan). The neoadjuvant targeted therapy regimen was cetuximab in 39 patients and bevacizumab in 25 patients. Both groups were comparable and eligible for efficacy, toxicity, and survival analyses. After reviewing the literature, mCRC typically develops metachronously [3,5,6], but most mCRC patients developed synchronously in our study.

**Table 1:** Baseline characteristics of 64 metastatic colorectal cancer patients

	Patients No. (%) (N = 64)
Age, years Mean + SD (range) ≧65 years <65 years	60 (30–83) 28 (43.8) 36 (56.3)
Gender Male Female	40 (62.5) 24 (37.5)
Chemotherapy/targeted therapy cycle =6 ≥7	28 (43.8) 36 (56.2)
Metastectomy location Liver Lung Ovary	46 (71.9) 15 (23.4) 3 (4.7)
Primary lesion site Right-side Left-side	7 (10.9) 57 (89.1)
Synchronous/Metachronous Synchronous Metachronous	36 (56.3) 28 (43.8)
Operation Operation time (min, medium + SD) (range) Estimated blood loss (ml, medium + SD) (range)	150 + 109.73 (50–645) 100 + 154.73 (5–700)
*RAS* status Wild Mutant	53 (82.8) 11 (17.2)
Pre-treatment CEA (ng/ml) ≧5 <5 Post-treatment CEA (ng/ml) ≧5 <5	45 (70.3) 19 (29.7) 28 (43.8) 36 (56.3)
Type of targeted therapy Cetuximab Bevacizumab	39 (60.9) 25 (39.1)
Efficacy Response (CR + PR) Non-response (SD + PD)	47 (73.4) 17 (26.6)
Progression Yes No	35 (54.7) 29 (45.3)
Survival Yes No	41 (64.1) 23 (35.9)
Grade III or more adverse effect Yes No	10 (15.6) 54 (84.4)

Note: CEA = carcinoembryonic antigen; CR = complete response; PR = partial response; SD = stable disease; PD = progressive disease.

### Patients’ demographics

The most common site of distant metastases was the liver (71.9%), followed by the lung (23.4%) and ovary (4.7%). The primary tumor was located in the right-side colon (7 patients, 10.9%) and left-side colon (57 patients, 89.1%). For metastasectomy, the mean operation time was 150 ± 109.73 min (range, 50–645 min), and the estimated blood loss was 100 ± 154.73 mL (range 5–700 mL). Fifty-three patients (82.8%) had *RAS* wild-type mCRC, and 11 (17.2%) had *RAS* mutant-type mCRC. A favorable tumor response (CR or PR) was observed in 47 of 64 (73.4%) patients.

### Correlation between cycle numbers and clinicopathological features

The correlation between the cycle numbers of chemotherapy/targeted therapy and clinicopathologic features in the 64 patients with mCRC is summarized in Table 2. No significant association was observed between these two neoadjuvant treatment modalities and baseline clinicopathological features, including age, sex, metastasectomy location, primary tumor location, synchronous/metachronous, *RAS* status, pretreatment serum CEA level, posttreatment serum CEA level, targeted agents, and adverse events of grade III or more (all *p* > 0.05). However, shorter operation time, lower estimated blood loss, higher response rate, lower progression rate, and favorable survival were observed in ≥7-cycle group (all *p* < 0.05).

**Table 2:** Correlation between cycle numbers of neoadjuvant chemotherapy/targeted therapy and clinicopathologic features in 64 metastatic colorectal cancer patients

	Total	Cycle = 6	Cycle ≥ 7	Chi square/T statistic	*p*-value
	N = 64 (%)	N = 28 (%)	N = 36 (%)		
Age, years ≧65 years <65 years	28 (43.8) 36 (56.2)	12(42.9) 16 (57.1)	16 (44.4) 20 (55.6)	0.016	1.000
Gender Male Female	40 (62.5) 24 (37.5)	19 (67.9) 9 (32.1)	21 (58.3) 15 (41.7)	0.610	0.603
Metastectomy location Liver Lung Ovary	46 (71.9) 15 (23.4) 3 (4.7)	23 (82.1) 5 (17.9) 0	23 (63.9) 10 (27.8) 3 (8.3)	3.725	0.155
Primary tumor location Right-side Left-side	7 (10.9) 57 (89.1)	3 (10.7) 25 (89.3)	4 (11.1) 32 (88.9)	0.003	1.000
Synchronous/Metachronous Synchronous Metachronous	36 (56.2) 28 (43.8)	18 (64.3) 10 (35.7)	18 (50.0) 18 (50.0)	1.306	0.314
Operation Operation time (min, medium + SD) Estimated blood loss (ml, medium + SD)	150 + 109.73 100 + 154.73	200 + 137.45 200 + 199.28	160 + 80.67 90 + 116.32	2.271 2.429	0.027* 0.019*
*RAS* status Wild Mutant	53 (82.8) 11 (17.2)	22 (78.6) 6 (21.4)	31 (86.1) 5 (13.9)	0.6290	0.513
Pre-treatment CEA (ng/ml) ≧5 <5 Post-treatment CEA (ng/ml) ≧5 <5	45 (70.3) 19 (29.7) 28 (43.8) 36 (56.2)	19 (67.9) 9 (32.1) 15 (53.6) 13 (46.4)	26 (72.2) 10 (27.8) 13 (36.1) 23 (63.9)	0.144 1.951	0.786 0.207
Targeted agents Cetuximab Bevacizumab	39 (60.9) 25 (39.1)	17 (60.7) 11 (39.3)	22 (61.1) 14 (38.9)	0.001	1.000
Efficacy Response (CR + PR) Non-response (SD + PD)	47 (73.4) 17 (26.6)	15 (53.6) 13 (46.4)	32 (88.9) 4 (11.1)	10.071	0.004*
Progression Yes No	35 (54.7) 29 (45.3)	20 (71.4) 8 (28.6)	15 (41.7) 21 (58.3)	5.630	0.024*
Survival Yes No	41 (64.1) 23 (35.9)	13 (46.4) 15 (53.6)	28 (77.8) 8 (22.2)	6.723	0.017*
Grade III or more adverse effect Yes No	10 (15.6) 54 (84.4)	2 (7.1) 26 (92.9)	8 (22.2) 28 (77.8)	2.717	0.165

Notes: CEA = carcinoembryonic antigen; CR = complete response; PR = partial response; SD = stable disease; PD = progressive disease.

*p-value < 0.05.

### Correlation between response and clinicopathological features

Univariate analysis revealed significant differences in chemotherapy/targeted therapy cycles and pretreatment serum CEA levels (both *p* < 0.05) between patients with a favorable response and those without a favorable response (Table 3). No significant differences were observed in age, sex, metastasectomy location, synchronous/metachronous, *RAS* status, posttreatment serum CEA level, type of targeted therapy, and adverse events of grade III or more (all *p* > 0.05).

**Table 3:** Analysis of predictors of response status in 64 metastatic colorectal cancer patients

	Response	Non-response	*p*-value
**Variables**	**(n = 47) (%)**	**(n = 17) (%)**	
Age, years <65 years *vs*. ≧65 years)	21 (44.7)/26 (55.3)	7 (41.2)/10 (58.8)	1.000
Gender (female *vs*. male)	28 (59.6)/19 (40.4)	12 (70.6)/5 (29.4)	0.562
Chemotherapy/targeted therapy cycles (≥7 *vs*. =6)	32 (68.1)/15 (31.9)	4 (23.5)/13 (76.5)	0.004*
Metastectomy location Liver Lung Ovary	31 (66.0) 13 (27.7) 3 (6.4)	15 (88.2) 2 (11.8) 0	0.193
Synchronous/Metachronous Synchronous Metachronous	27 (57.4) 20 (42.6)	9 (52.9) 8 (47.1)	0.782
*RAS* status Wild Mutant	42 (79.4) 5 (10.6)	11 (64.7) 6 (35.3)	0.054
Pre-treatment CEA (ng/ml) ≧5 <5	37 (78.7) 10 (21.3)	8 (47.1) 9 (52.9)	0.028*
Post-treatment CEA (ng/ml) ≧5 <5	20 (42.6) 27 (57.4)	8 (47.1) 9 (52.9)	0.782
Targeted agents Cetuximab Bevacizumab	31 (66.0) 16 (34.0)	8 (47.1) 9 (52.9)	0.246
Grade III or more adverse effect Yes No	8 (17.0) 39 (83.0)	2 (11.8) 15 (88.2)	1.000

Notes: CEA = carcinoembryonic antigen.

*p-value < 0.05.

### Correlation between overall survival, progression and clinicopathological features

Among the 64 patients, 41 (64.1%) patients survived (the data of 6 of these patients were censored), and 23 did not. Univariate analysis revealed significant differences in chemotherapy/targeted therapy cycles and pretreatment serum CEA levels (both *p* < 0.05) between patients who survived and those who did not (Table 4). No significant differences were noted in age, sex, metastasectomy location, synchronous/metachronous, *RAS* status, posttreatment serum CEA level, targeted agents, and adverse events of grade III or more (all *p* > 0.05).

**Table 4:** Analysis of predictors of overall survival in 64 metastatic colorectal cancer patients

	Survival	Non-survival	*p*-value
**Variables**	**(n = 36^#^) (%)**	**(n = 23^#^) (%)**	
Age, years <65 years *vs*. ≧ 65 years)	24 (66.7)/12 (33.3)	11 (47.8)/12 (52.2)	0.085
Gender (female *vs*. male)	14 (38.9)/22 (61.1)	9 (39.1)/14 (60.9)	0.702
Chemotherapy/targeted therapy cycles (≥7 *vs*. =6)	27 (75.0)/9 (25.0)	8 (34.8)/15 (65.2)	0.002*
Metastectomy location Liver Lung Ovary	26 (72.2) 7 (19.4) 3 (8.3)	16 (69.6) 7 (30.4) 0	0.533
Synchronous/Metachronous Synchronous Metachronous	20 (55.6) 16 (44.4)	12 (52.2) 11(47.8)	0.520
*RAS* Status Wild Mutant	29 (80.6) 7 (19.4)	21 (91.3) 2 (8.7)	0.210
Pre-treatment CEA (ng/ml) ≧5 <5	20 (55.6) 16 (44.4)	20 (87.0) 3 (13.0)	0.012*
Post-treatment CEA (ng/ml) ≧5 <5	12 (33.3) 24 (66.7)	13 (56.5) 10 (43.5)	0.161
Type of targeted therapy Cetuximab Bevacizumab	24 (66.7) 12 (33.3)	12 (52.2) 11(47.8)	0.538
Grade III or more adverse effect Yes No	5 (13.9) 31 (86.1)	3 (13.0) 20 (87.0)	0.941

Notes: CEA = carcinoembryonic antigen.

^#^Number of censor case: 5.

*p-value < 0.05.

After metastasectomy, disease progression was observed in 35 (54.7%) of 64 patients. Univariate analysis revealed a significant difference in chemotherapy/targeted therapy cycles (*p* = 0.024) between patients with and without disease progression (Table 5). No significant differences were observed in age, sex, metastasectomy location, synchronous/metachronous, *RAS* status, pretreatment serum CEA level, posttreatment serum CEA level, targeted agents, and adverse events of grade III or more (all *p* > 0.05).

**Table 5:** Analysis of predictors of progression in 64 metastatic colorectal cancer patients

	Progression	Non-progression	*p*-value
Variables	(n = 35) (%)	(n = 29) (%)	
Age, years <65 years *vs*. ≧65 years)	19 (54.3)/16 (45.7)	17 (58.6)/12 (41.4)	0.803
Gender (female *vs*. male)	14 (40.0)/21 (60.0)	10 (34.5)/19 (65.5)	0.796
Chemotherapy/targeted therapy cycles (≥7 *vs*. =6)	15 (42.9)/20 (57.1)	21 (72.4)/8 (27.6)	0.024*
Metastectomy location Liver Lung Ovary	26 (74.3) 8 (22.9) 1 (2.9)	20 (69.0) 7 (24.1) 2 (6.9)	0.731
Synchronous/Metachronous Synchronous Metachronous	20 (57.1) 15 (42.9)	16 (55.2) 13 (44.8)	1.000
*RAS* Status Wild Mutant	26 (74.3) 9 (25.7)	27 (93.1) 2 (6.9)	0.093
Pre-treatment CEA (ng/ml) ≧5 <5	26 (74.3) 9 (25.7)	19 (65.5) 10 (34.5)	0.584
Post-treatment CEA (ng/ml) ≧5 <5	17 (48.6) 18 (51.4)	11 (37.9) 18 (62.1)	0.454
Targeted agents Cetuximab Bevacizumab	21 (60.0) 14 (40.0)	18 (62.1) 11 (37.9)	1.000
Grade III or more adverse effect Yes No	6 (17.1) 29 (82.9)	4 (13.8) 25 (86.2)	1.000

Note: CEA = carcinoembryonic antigen.

*p-value < 0.05.

### Toxicity

Postoperative complications in 64 patients with mCRC who underwent metastasectomy after chemotherapy/targeted therapy are presented in Table 6. Few patients developed postoperative complications. No significant differences were noted in postoperative complications (surgical and nonsurgical) between the two groups (all *p* > 0.05).

**Table 6:** Postoperative complications in 64 patients with metastatic colorectal cancer patients undergoing metastectomy after chemotherapy/targeted therapy

Complications	Cycle = 6 N = 28 (%)	Cycle ≥ 7 N = 36 (%)	*p*-value
Surgical Postoperative bleeding Intra-abdominal infection/abscess Ileus Wound infection	9 (32.1) 2 (7.1) 2 (7.1) 2 (7.1) 3 (10.7)	7 (19.4) 1 (2.8) 2 (5.6) 2 (5.6) 2 (5.6)	0.220
Non-surgical Pulmonary complication Urinary tract infection Urine retention Myocardial infarction Acute kidney injury	8 (28.6) 2 (7.1) 2 (7.1) 2 (7.1) 1 (3.6) 1 (3.6)	7 (19.4) 2 (5.6) 2 (5.6) 2 (5.6) 0 1 (2.8)	0.218

### Survival analysis

**Figure 2:**
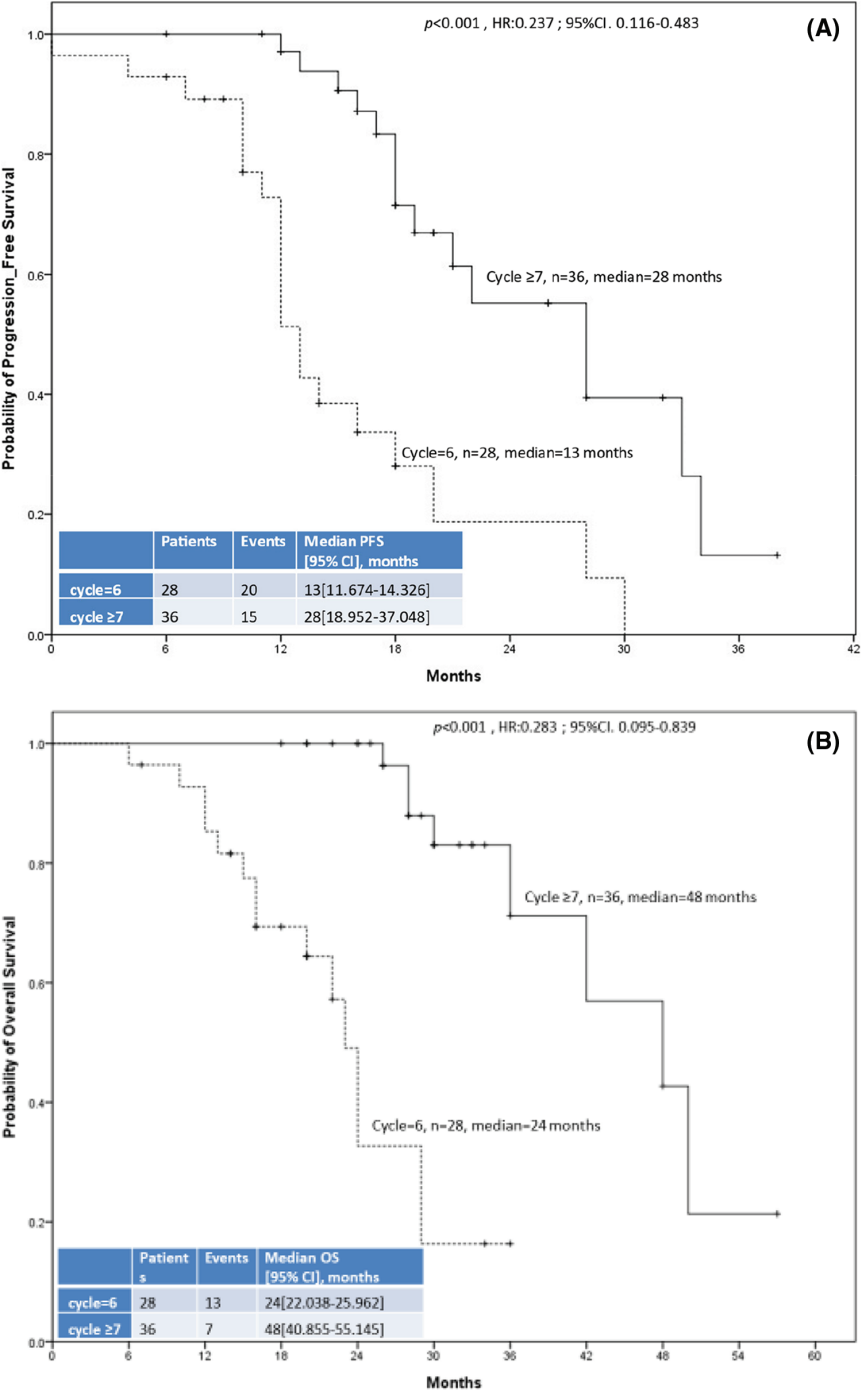
The 64 enrolled patients with metastatic colorectal cancer included 28 who received 6 cycles of neoadjuvant chemotherapy/targeted therapy (dashed line) and 36 who received ≥7 cycles (solid line). (A) Progression-free survival. (B) Overall survival.

Fig. 2 displays the PFS and OS of the two groups. The median OS and PFS were 24 months (95% CI: 22.038–25.962) and 13 months (95% CI: 11.674–14.326), respectively, in the 6-cycle group and 48 months (95% CI: 40.855–55.145) and 28 months (95% CI: 18.952–37.048), respectively, in the ≥7-cycle group. Thus, ≥7 cycles of neoadjuvant therapy led to better survival than 6 cycles (both *p* < 0.001).

### Proportional Hazards Assumption in Cox Regression Analyses

Table 7 presents the proportional hazard assumption in univariate and multivariate Cox regression analyses of predictors of progression and survival in 64 patients with mCRC. PFS was significantly shorter in the 6-cycle group than in the ≥7-cycle group (HR: 0.180; 95% CI, 0.073–0.440; *p* < 0.001), and the patients with *RAS* wild-type mCRC had better PFS than those with *RAS* mutant-type mCRC (HR: 9.787; 95% CI, 2.518–38.0333; *p* = 0.001). Moreover, OS was significantly longer in the ≥7-cycle group than in the 6-cycle group, and patients in the 6-cycle group had a shorter OS than those in the ≥7-cycle group (HR: 0.036; 95% CI, 0.008–0.159; *p* < 0.001).

**Table 7:** The proportional hazard assumption in univariate and multivariate Cox regression analysis of predictors of progression-free survival and overall survival in 64 metastatic colorectal cancer patients

	Progression-free survival				Overall survival			
Variables	Univariate analysis HR (95% CI)	*p*-value	Multivariate analysis HR (95% CI)	*p*-value	Univariate analysis HR (95% CI)	*p*-value	Multivariate analysis HR (95% CI)	*p*-value
Age, years (<65 *vs*. ≧65)	0.779 (0.386–1.575)	0.487	0.979 (0.433–2.217)	0.960	1.674 (0.720–3.890)	0.231	2.377 (0.943–5.991)	0.066
Gender (female *vs*. male)	1.380 (0.685–2.779)	0.372	2.207 (0.871–4.715)	0.101	1.065 (0.454–2.498)	0.885	1.838 (0.614–5.495)	0.276
Chemotherapy/targeted therapy cycles (=6 *vs*. ≥7)	0.237 (0.116–0.483)	<0.001*	0.180 (0.073–0.440)	**<0.001** ^*^	0.094(0.033–0.269)	<0.001*	0.036 (0.008–0.159)	<0.001*
Metastectomy location Liver Lung Ovary	0.577 (0.276–1.207) 0.834 (0.545–1.907) 1.00	0.144 0.285	0.595 (0.185–1.301) 0.912 (0.512–1.952) 1.00	0.288 0.355	1.154 (0.475–2.805) 1.015 (0.501–2.521) 1.00	0.752 0.683	1.254 (0.452–3.012) 1.352 (0.522–2.652)	0.652 0.412
Synchronous/Metachronous Synchronous Metachronous	1.125 (0.798–1.586) 1.00	0.500	0.999 (0.418–2.389) 1.00	0.998	1.064 (0.693–1.633) 1.00	0.778	0.499 (0.175–1.428) 1.00	0.195
*RAS* Status Wild Mutant	2.879 (1.297–6.390) 1.00	**0.009** ^*^	9.787 (2.518–38.033) 1.00	**0.001** ^*^	0.431 (0.099–1.880) 1.00	0.263	0.332 (0.065–1.694) 1.00	0.185
Pre-treatment CEA (ng/ml) ≧5 <5	1.195 (0.556–2.565) 1.00	0.644	1.051 (0.378–2.918) 1.00	0.924	2.122 (0.620–7.265) 1.00	0.231	1.529 (0.339–6.9.4) 1.00	0.581
Post-treatment CEA (ng/ml) ≧5 <5	1.005 (1.001–1.008) 1.00	0.025*	1.842 (0.795–4.265) 1.00	0.154	1.729 (0.741–4.027) 1.00	0.204	1.452 (0.469–4.495) 1.00	0.517
Type of targeted therapy Cetuximab Bevacizumab	0.958(0.683–1.346) 1.00	0.806	2.083 (0.734–5.932) 1.00	0.168	0.944 (0.612–1.456) 1.00	0.795	0.314 (0.103–0.955) 1.00	**0.041** ^*^
Grade III or more adverse effect Yes No	0.976 (0.627–1521) 1.00	0.916	0.387 (0.130–1.158) 1.00	0.090	1.084 (0.586–2.003) 1.00	0.798	0.545 (0.125–2.371) 1.00	0.419

Notes: CEA = carcinoembryonic antigen; HR = Hazard Ratio.

*p-value < 0.05.

## Discussion

CRC is one of the most common cancers and one of the leading global cause of cancer death. Our previous study identified CRC as one of the leading malignant tumors in Taiwan [12]. Timely radical metastasectomy is key for favorable outcomes. For mCRC patients with synchronous metastases, the decision to proceed with neoadjuvant chemotherapy/targeted therapy is straightforward. Neoadjuvant chemotherapy/targeted therapy is already being practiced in many institutions in patients with CRC undergoing metastasectomy, although the principle of the cycles of chemotherapy/targeted therapy has not yet been formally validated [9].

In recent years, a multidisciplinary treatment approach, including neoadjuvant chemotherapy and targeted therapy, has emerged for mCRC, resulting in increased curability and improved survival [6,11,12,15]. We determined the effects of increasing the number of cycles of neoadjuvant chemotherapy/targeted therapy on survival and clinical outcomes of patients with mCRC undergoing major metastasectomy. Our findings indicated that this strategy did not cause postoperative complications but was associated with improved efficacy and survival, with comparable adverse events.

Neoadjuvant therapy may increase the likelihood of completing multimodality therapy, particularly when surgical management is associated with significant morbidity and complications that may preclude timely adjuvant therapy. The advantages of neoadjuvant chemotherapy/targeted therapy for patients with mCRC include surgical field sterilization, potential reduction of the risk of tumor dissemination at resection, and comparable surgical morbidity [9,12].

The COIN trial was a randomized trial that examined the effects of adding cetuximab, an anti-EGFR monoclonal antibody, to the standard oxaliplatin-based chemotherapy regimens as a first-line treatment for patients with advanced CRC; the results revealed that the addition of cetuximab did not affect OS or PFS but increased tumor response rates in patients with the wild-type *KRAS* genotype [20]. The addition of bevacizumab, an anti-VEGF monoclonal antibody, to standard chemotherapy regimens may increase response rates and median OS among patients with mCRC [21]. Moreover, determination of the *UGT1A1* polymorphism as guidance for irinotecan dose escalation in patients with mCRC can achieve more favorable clinical outcomes without significantly increased toxicities [22].

Patients who underwent curative metastasectomy exhibited better outcomes than those who did not undergo surgery. Low resectability of metastasis is the main cause of poor prognosis in patients with mCRC who cannot undergo curative surgery [23]. In patients whose metastases become resectable after neoadjuvant therapy, it could be helpful to know if metastasectomy should be performed as soon as possible or after one or more cycles of chemotherapy/targeted therapy to allow for more tumor shrinkage. Determination of the type of cycle of neoadjuvant chemotherapy/targeted therapy is useful.

The advantages of neoadjuvant therapy may be the most pronounced in specific patient subsets. Although most studies have reported favorable OS in patients with mCRC following neoadjuvant chemotherapy/targeted therapy, the factors associated with efficacy, safety, and survival differ [15,24,25]. Currently, no consensus exists on which neoadjuvant protocol or the number of cycles that is superior for treating patients with mCRC. Trial results are controversial, resulting in strong interinstitutional differences concerning chemotherapy/targeted therapy sequences for treating patients with mCRC. The optimal cycle numbers of chemotherapy/targeted therapy for metastasectomy for patients with mCRC remains uncertain [26,27].

Our results revealed significant associations between cycles of neoadjuvant chemotherapy/targeted therapy and efficacy/survival outcomes. In particular, the ≥7-cycle group had shorter operation time, lower estimated blood loss, higher response rate, lower disease progression rate, and longer survival than the 6-cycle group. In addition, 47 (73.4%) of the 64 patients were categorized into the response group and the remaining 17 patients (26.6%) into the nonresponse group. Moreover, number of neoadjuvant therapy cycles and the pretreatment serum CEA level were independent predictors of response and survival, and the number of neoadjuvant therapy cycles was as an independent predictor of disease progression. Overall, both PFS and OS were longer in the ≥7-cycle group, and favorable PFS was also noted in patients with *RAS* wild-type mCRC. According to results from the Fire-3 trial, OS and objective response of mCRC patients receiving cetuximab were greatly superior to bevacizumab in patients with elevated CEA, while this effect was markedly lower and lost statistical significance in patients with low CEA [28]. FOLFIRI/cetuximab exhibited a significantly superior objective response rate in patients with high CEA in contrast to patients with low CEA [28]. However, we did not observe any difference in the morbidity rate between the two treatment groups, with tolerable toxicity and safety. Together, these findings support the administration of ≥7 cycles of chemotherapy/targeted therapy in patients with mCRC before metastasectomy without significantly increased surgical complications.

This study has some limitations. Our study was a retrospective single-center study with a limited sample size. Large-scale prospective, randomized studies with careful patient monitoring are required to validate our findings. More specially designed studies and reliable biological indicators of real functional status are required to properly select patients for multimodal treatment. The results of such studies could be used to demonstrate the efficacy of treatment for mCRC.

The decision for an optimal neoadjuvant treatment strategy for patients with mCRC metastasectomy remains complex and controversial. Our findings support the administration of ≥7 cycles of neoadjuvant chemotherapy/targeted therapy in these patients, as indicated by more favorable OS and PFS, better response rate, and no significant increase in toxicity compared with the administration of 6 cycles. Future multicenter prospective randomized trials are warranted to validate our results.

## Data Availability

The datasets supporting the conclusions of this manuscript are included within the article. Please contact author for raw data requests.
